# Relapsing polychondritis associated with bilateral stapes footplate fixation: a case report

**DOI:** 10.4076/1752-1947-3-8496

**Published:** 2009-09-09

**Authors:** Yohanna M Takwoingi

**Affiliations:** 1 Department of Otolaryngology, Head and Neck Surgery, City Hospital, Dudley Road, Birmingham, UK

## Abstract

**Introduction:**

Relapsing polychondritis is a rare multisystem autoimmune disease of uncertain etiology characterized by recurrent episodes of inflammation and progressive destruction of cartilaginous tissues. Its respiratory, cardiovascular, renal and neurological complications are life-threatening, and it is thus important to recognize the disease and its complications early. Relapsing polychondritis may follow a slowly evolving or rapidly progressive course.

**Case presentation:**

The case of a 39-years-old Caucasian woman with a three-year history of recurrent bilateral chondritis of the auricles, nasal chondritis, seronegative polyarthritis and dermatitis is reported. She had an associated bilateral stapedial fixation and one side was successfully operated on. She also had a large septal perforation involving both the cartilaginous and bony parts. The patient first presented with severe cutaneous inflammation when she was only one month old, and so this is an illustrative case of relapsing polychondritis that slowly evolved over many years.

**Conclusions:**

Relapsing polychondritis is still a relatively uncommon condition, which explains why there is often a delayed diagnosis of the disease. It is usually difficult to examine tympanic membranes in cases of relapsing polychondritis, and, therefore stapes fixation should also be suspected when there is an associated conductive hearing loss.

## Introduction

Relapsing polychondritis (RP) is an uncommon connective tissue disease characterized by recurrent episodes of inflammation and progressive destruction of cartilaginous tissues. The elastic cartilage of the ears and nose, the hyaline cartilage of the peripheral joints, the vertebral fibrocartilage and the tracheobronchial cartilage, as well as the proteoglycan-rich structures of the eye, heart, blood vessels or inner ear may all be affected [1]. In most patients, RP manifests in a fluctuating but progressive course that eventually results in a significant shortening of life expectancy. The average age of the disease's onset is at 47 years. Cochlear and vestibular involvement commonly accounts for sensorineural deafness [2,3]. Previous studies show the occurrence of conductive deafness due to stenosis of the external auditory canal or otitis media with effusion probably as a result of Eustachian tube involvement [3].

The case of a patient with associated bilateral stapes fixation who developed symptoms at a very young age is reported here.

## Case presentation

A 39-years-old Caucasian woman with a three-year history of recurrent swellings of both pinnae presented to our hospital's ENT department. Two weeks before presenting to the hospital, she suffered acute exacerbations that led to the obstruction of her left external auditory meatus. The swelling was painless and her main problem was diminished hearing as a result of the sudden occlusion of her external auditory canal. Although no positive family history could be found, the patient's personal medical history revealed that she suffered from generalized eczema and asthma during childhood. Her hearing was also impaired and she used hearing aids between the ages of one and 16 years.

Her clinical records revealed that she first presented to a dermatologist due to intertrigal dermatitis when she was only 26 days old. By the time she was four months old, her whole body was covered with inflammation. She developed asthma at the age of four years and was diagnosed to have an eczema-asthma syndrome (ichthyosis and eczema). At the age of 11, she developed polyarthritis that affected her large and small joints. Rheumatoid arthritis was ruled out after due investigations, but she continued to receive treatment for seronegative polyarthritis.

**Figure 1:**
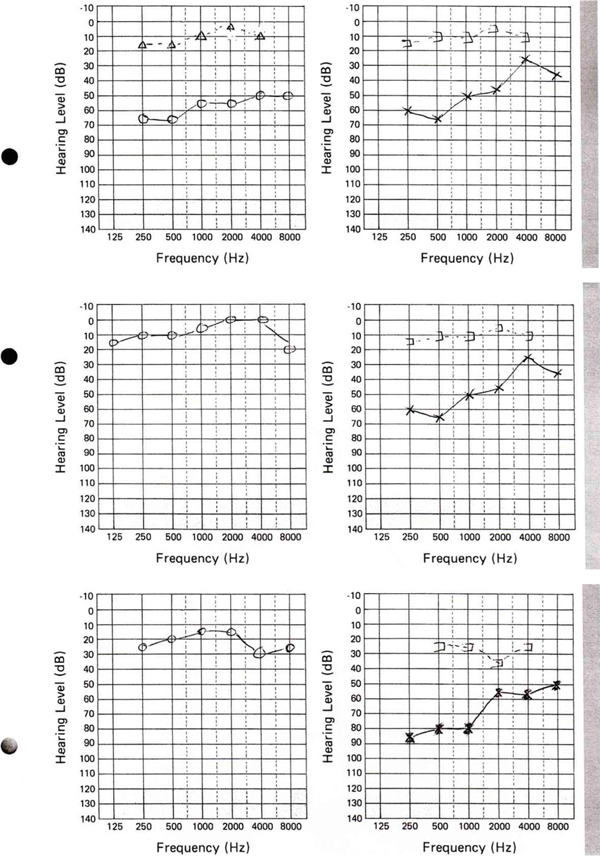
****(A)** Preoperative audiogram showing identical conductive hearing loss**. **(B)** Postoperative audiogram showing normal hearing in the operated ear. **(C)** Audiogram at 23 years after right stapedectomy.

When the patient was 14 years old she presented to ENT with nasal obstruction and a large septal perforation. An audiogram examination showed that the patient had a bilateral conductive loss of hearing with an average of 35 decibels of air-bone gap across the 0.5, 1, 2 and 4KHz speech frequencies (Figure 1A). Tympanograms were peaked and showed a normal pressure range. Ossicular chain malformation was suspected and so her right ear was explored. A fixed stapes was discovered and a partial stapedectomy was carried out, which restored her normal hearing (Figure 1B).

Ten years later, she was offered surgical stapedectomy to improve hearing in her left ear but the patient declined this, as she was content with the normal hearing in one ear. The patient has maintained normal hearing in her right ear until she was 39-years-old (Figure 1C).

**Figure 2:**
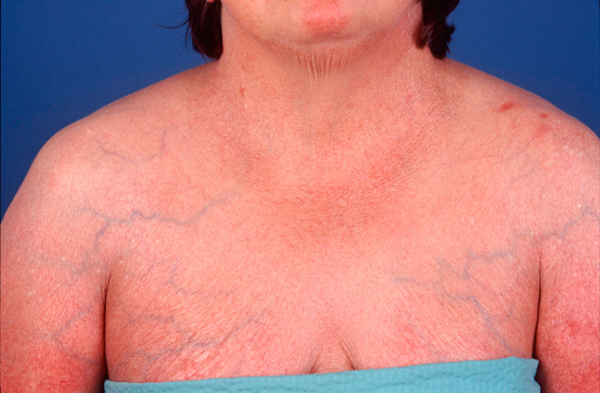
**A photograph showing generalized cutaneous inflammation**.

**Figure 3:**
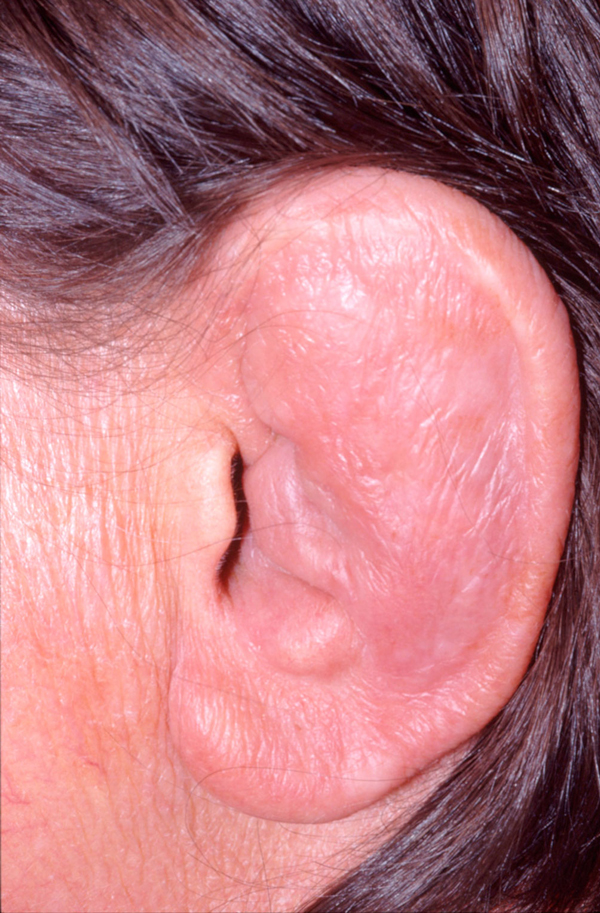
**A photograph of the pinna showing auricular chondritis, with the earlobe spared of any inflammation**.

**Figure 4:**
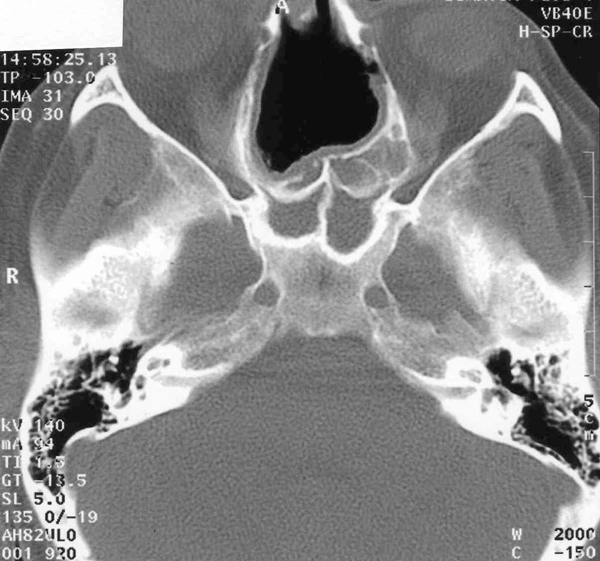
**CT scan of the sinuses demonstrating a large septal perforation**.

A clinical examination of the patient revealed that she had thick, dry, scaly skin and scalp. Serous discharge from the skin folds and the scalp was also discovered. There was also a marked generalized telangiectasia (Figure 2). Both auricles of the patient were chronically inflamed with the loss of normal contours but her earlobes bore no such inflammation (Figure 3). A cystic swelling measuring about 3 × 4 cm in diameter filled the patient's left concha, which obstructed the external meatus. Her tympanic membranes were not visible. The patient had rhinolalia and her nose had a large septal perforation of both the cartilaginous and bony nasal septum (Figure 4). The lateral walls of the nasal cavity were crusty and both middle and superior turbinates were atrophic and unidentifiable.

Biochemical analysis and microbiological study of aspirate from the cystic swelling were unremarkable. Full blood count, erythrocyte sedimentation rate, urea, and electrolyte were all normal. Anti-neutrophil cytoplasmic antibody (c-ANCA), antinuclear antibodies, smooth muscles antibodies, and Venereal Disease Research Laboratory (VDRL) and treponema palladium haemagglutination (TPHA) tests for syphilis were all negative. Rheumatoid arthritis screen and autoantibody screen were also negative. Biopsies of the patient's left auricular cartilage, nasal septal cartilage and the lateral walls of the nose revealed a chronic non-specific inflammation.

Based on her clinical features at presentation and her medical history, a clinical diagnosis of relapsing polychondritis was made, the patient having exhibited the following features: 1) recurrent bilateral auricular chondritis; 2) non-erosive seronegative polyarthritis; 3) chondritis of nasal cartilage; and 4) cutaneous inflammation. She was subsequently started on treatment. Seven years later, the activity of the patient's disease is under control although there is a significant irreversible damage to the affected organs. She was initially treated with high dose prednisolone and cyclophosphamide and is now on maintenance doses of prednisolone and azathioprine.

## Discussion

Relapsing polychondritis is an uncommon, chronic multisystem disorder characterized by recurrent episodes of inflammation of the cartilaginous tissues that often leads to progressive destruction. The disease can be life-threatening, debilitating and difficult to diagnose [4]. The clinical features of the disease have been well-defined and the diagnostic criteria outlined by McAdam et al. [5] are generally accepted. The diagnosis of relapsing polychondritis is said to be conclusive if a patient has at least three of the six features of the disease, namely: 1) bilateral auricular chondritis; 2) non-erosive seronegative inflammatory arthritis; 3) nasal chondritis; 4) ocular inflammation; 5) respiratory tract chondritis; and 6) audiovestibular damage with or without histological confirmation.

Meanwhile, Damiani and Levine [2]. Modified the above criteria to be as follows: three or more of McAdam's signs with a positive histology, and an involvement of three or more separate anatomic locations with response to steroids and/or dapsone. All types of cartilage may be involved. RP can also cause an inflammation of other proteoglycan-rich structures such as the eye, the heart, blood vessels and the inner ear [4].

The patient described in this report exhibited the following signs: recurrent bilateral auricular chondritis; non-erosive, seronegative polyarthritis; and chondritis of the nasal cartilage. Another feature in this patient is generalized skin inflammation. Classified as a minor sign in RP, skin inflammation was also her initial symptom when she first presented as an infant and has continued almost throughout her life.

This case report underscores the diagnostic difficulty commonly encountered in cases of RP. The patient described here had seen various specialists for features and conditions suggestive of RP from a very young age. However, she was only finally diagnosed with the disease when she was 39-years-old.

The most common presenting feature in RP is auricular chondritis, which affects up to 89% of cases and is bilateral in 95% of patients reported [2,5]-[7]. RP often causes pain, redness and swelling of the pinna, and sparing of the earlobe (Figure 2). The known average age of onset of the disease is 47 years with a range of 13 to 84 years [3]-[5]. Damiani et al. reported a case involving a three-year-old whose mother also suffered from RP at the time of pregnancy [2]. Apart from rare cases, no familial predisposition has been noted. It is said that less than 10% of cases are seen in adolescents and children [8]. The patient described in this report developed symptoms when she was still an infant, which is an unusually early age to develop the disease.

There is no diagnostic laboratory test to determine RP. Antinuclear antibodies generally yield negative results unless there is already an associated connective tissue disease [4]. Low titres of ANCA, either diffuse or perinuclear, were noted to be present in serum samples of 24% of patients studied [2]. However, C-ANCA and other laboratory tests were negative in this patient. No biopsy finding is pathognomonic for RP and non-specific inflammation is often found [4,9]. Biopsy is therefore to be discouraged as it only adds to the mutilating nature of the disease. Such non-specific findings from biopsies are also common in other chronic conditions such as Wegener's granulomatosis [10]. Some authors have also emphasized that there is a significant correlation between elevated ESR and disease activity [2,4]. However, this was not observed in this patient.

Both conductive and neurosensory deafness have been described in RP. Cochlear and vestibular involvement commonly accounts for the occurrence of sensorineural deafness [2,3,7]. The mechanism of inner ear involvement is unclear, but there are suggestions that this may happen as a result of inflammatory response. The presence of anti-labyrinthine antibodies in a patient with RP has been previously identified [11]. Conductive hearing loss is thought to be due to either the closure of the Eustachian tube from inflammation in the cartilaginous wall leading to serous otitis media, auricular cartilage collapse or oedema of the canal [1,4,6]. The occurrence of auricular cartilage chondritis and the involvement of the external auditory canal, however, often mean that it is not possible to properly examine the tympanic membranes to be able to draw any meaningful conclusions. The patient presented here had bilateral conductive impairment and stapes fixation was confirmed to be the cause of the impairment of the ear that was operated on. The other ear had identical audiometric features (Figure 1A).

The association between stapes fixation and RP has not been previously described in the literature. Nevertheless, although probably just coincidental in the patient described in this report, the presumptive diagnosis of serous otitis media in patients with collapsed external canals may well be inaccurate. The presence of stapes fixation should also be suspected in patients. Otosclerosis has a prevalence of 2% both in adult men and women patients [12]. Age at presentation of otosclerosis typically ranges from the teenage years to the late 40's, with individuals younger than 18 accounting for only 15% of documented cases [13,14]. This patient underwent stapedectomy at the age of 16 years.

Moreover, most studies in families support a pattern of autosomal dominant transmission with complete penetrance, but several studies have also reported sporadic cases of otosclerosis. The patient reported here did not give any family history that is suggestive of otosclerosis.

Chondritis due to RP can affect all types of cartilage, and so it is reasonable to suspect that the process of generalized chondritis may also involve chondrification of the fissula ante fenestram. Through the process of relapsing chondritis, modelling and remodelling of the surrounding bone with consequent stapes fixation is a possibility [15]. Since fragments from the stapes footplate were not submitted to histological examination, the fixation is best described as having an uncertain origin rather than due specifically to otosclerosis. This relationship has not been previously described and it is entirely possible that the patient reported here has two independent disease processes, and this coincidence is interesting because conductive hearing impairment has long been associated with RP.

Saddle nose deformity is commonly associated with nasal chondritis in this disease, and this feature is said to appear most often in females younger than 50 years old [3,4]. This patient has a large septal perforation involving both the cartilaginous and bony parts of the nose, yet no collapse was observed (Figure 4). Bone destruction may have been the result of neglect and secondary atrophy, but the involvement of bony septum in RP has not been previously reported.

Prognosis is linked to laryngeal, tracheal and cardiovascular involvements. Chondritis of the respiratory tract is one of the most serious complications of relapsing polychondritis and accounts for up to 50% of deaths due to RP. The respiratory tract is thought to be involved in up to 50% of documented cases, and so it is crucial to look for respiratory signs in order to offer appropriate treatment.

Cardiovascular deterioration is the second most frequent cause of death in patients with RP [4], and aortic and mitral regurgitation are the most common cardiovascular manifestations. This patient did not have symptoms related to any of these systems and thorough clinical examination and ECG tests yielded normal results. Echocardiography showed only a trivial reflux of the tricuspid valves. No subglottic involvement was encountered when she underwent examination under general anaesthesia. Earlier studies indicate survival rates between 70% at four years and 55% at 10 years of the disease's onset. In a recent study, a survival rate of 94% at 8 years may be due to improved medical and surgical management [4].

## Conclusions

RP is still relatively uncommon, which explains why the diagnosis is often delayed. The patient described in this report fulfilled the diagnostic criteria by having three major signs and one minor sign of cutaneous inflammation. She also first manifested her symptoms as an infant. Additionally, this is the first documented case of associated bilateral stapes fixation in RP. In the presence of conductive hearing loss in RP, stapes fixation should also be considered as a differential diagnosis. Although this is generally a rare condition, greater awareness especially among otolaryngologists will ensure early detection and early treatment.

## Abbreviations

c-ANCA: cytoplasmic antineutrophil cytoplasmic antibody; ENT: Ear, Nose and Throat; ESR: erythrocyte sedimentation rate; RP: relapsing polychondritis.

## Competing interests

The author declares that he has no competing interests.

## Consent

Written informed consent was obtained from the patient for publication of this case report and any accompanying images. A copy of the written consent is available for review by the Editor-in-Chief of this journal.
